# Supplementary material for: Universal salt-assisted assembly of MXene from suspension on polymer substrates
Source: Nat Commun. 2024 Nov 25;15:10027. doi: 10.1038/s41467-024-53840-y (PMC11589784; doi:10.1038/s41467-024-53840-y)
Supplement: Supplementary file 1 — Supplementary Information [file 41467_2024_53840_MOESM1_ESM.pdf]

Supplementary Information

Universal salt-assisted assembly of MXene from suspension on polymer substrates

Liang ZHAO, Lingyi BI, Jiayue HU, Guanhui GAO, Danzhen ZHANG, Yun LI, Aidan FLYNN, Teng ZHANG, Ruocun WANG, Xuemei M. CHENG, Ling LIU*, Yury GOGOTSI*, Bo LI*

*Corresponding authors. Email: ling.liu@temple.edu (LL), gogotsi@drexel.edu (YG), bo.li@villanova.edu (BL)

This PDF file includes:

Supplementary Figures 1 to 26,

Supplementary Tables 1 to 8

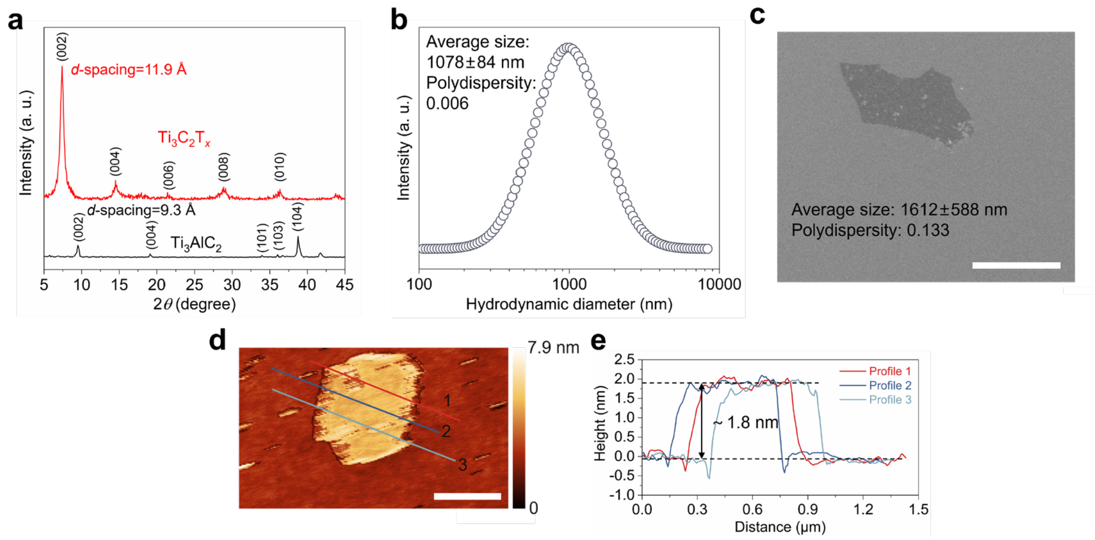

Supplementary Figure 1. Characterization of pristine $Ti_3C_2T_x$ nanosheets. a, X-ray diffraction (XRD) patterns of Ti_3AlC_2 MAX phase powder and $Ti_3C_2T_x$ nanosheet film. The intensity of (002) peak for $Ti_3C_2T_x$ nanosheet film is enhanced, and the position shifts towards lower 2θ compared with Ti_3AlC_2 MAX phase powder, demonstrating the successful etching and delamination[1]. The $Ti_3C_2T_x$ nanosheet film for XRD measurement is prepared by drop-casting on a glass slide. b, Representative dynamic light scattering (DLS) curve of $Ti_3C_2T_x$ nanosheet suspension (concentration: 0.01 mg mL^{-1}). The calculated average lateral size of $Ti_3C_2T_x$ nanosheets is ~1078 nm. The error bar is based on the standard deviation of 5 samples. c, Representative SEM image of monolayer $Ti_3C_2T_x$ nanosheet. Scale bar, 1 μm. The error bar is based on the deviation of 30 nanosheets. d, Atomic force microscope (AFM) image of a monolayer $Ti_3C_2T_x$ nanosheet. Scale bar, 500 nm. e, Height profiles in (d). The thickness of the monolayer $Ti_3C_2T_x$ nanosheet is ~1.8 nm due to water and other adsorbed species between the flake and the substrate, similar to the reported single-layer MXene flakes values[2].

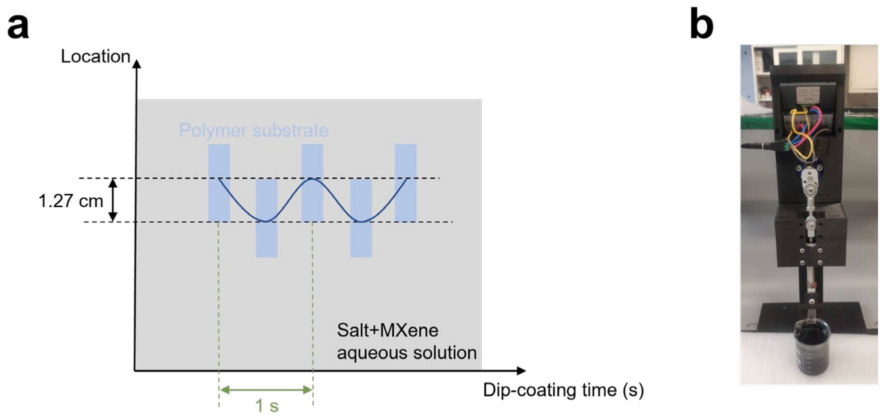

Supplementary Figure 2. Schematic of dip-coating process and corresponding parameters of SAA. a, The location of substrate vs. dip-coating time. b, The digital image of the customized dip coater. The rotating speed of the motor is 60 rpm, and the average dipping speed is 1.5m min^{-1}. The polymer substrates are always submersed in the salt-$Ti_3C_2T_x$ aqueous suspension for assembly. In addition, the shear field offered by the dipping process helps orient the MXene so that MXene nanosheets are well-aligned parallel to the substrate.

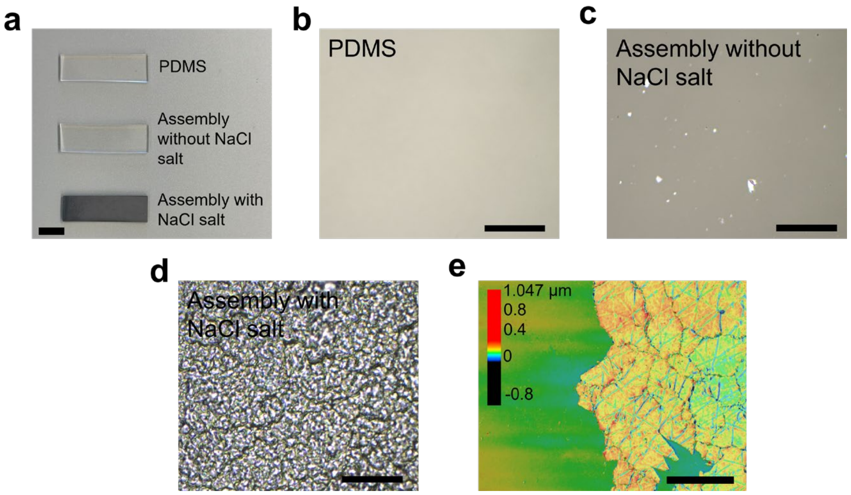

Supplementary Figure 3. $Ti_3C_2T_x$ nanosheet assembly on PDMS substrates with and without NaCl salt. a, Digital images of pure PDMS substrate and $Ti_3C_2T_x$ nanosheet assemblies on PDMS. Scale bar, 4 mm. b, Optical image of pure PDMS. c, Optical image of $Ti_3C_2T_x$ nanosheet assembly on PDMS without NaCl. Without NaCl salt addition to the $Ti_3C_2T_x$ nanosheet suspension, we failed to achieve full coverage and uniform assembly even though some nanosheets were assembled. d, Optical image of $Ti_3C_2T_x$ nanosheet assemblies on PDMS with NaCl. e, Optical profilometry map of $Ti_3C_2T_x$ nanosheet assemblies on PDMS with NaCl. Scale bars, 20 μm. The dip-coating assembly time is fixed to be 15 min, the $Ti_3C_2T_x$ nanosheet concentration is 5 mg mL^{-1}, and the NaCl salt concentration is 0.01 mol L^{-1}.

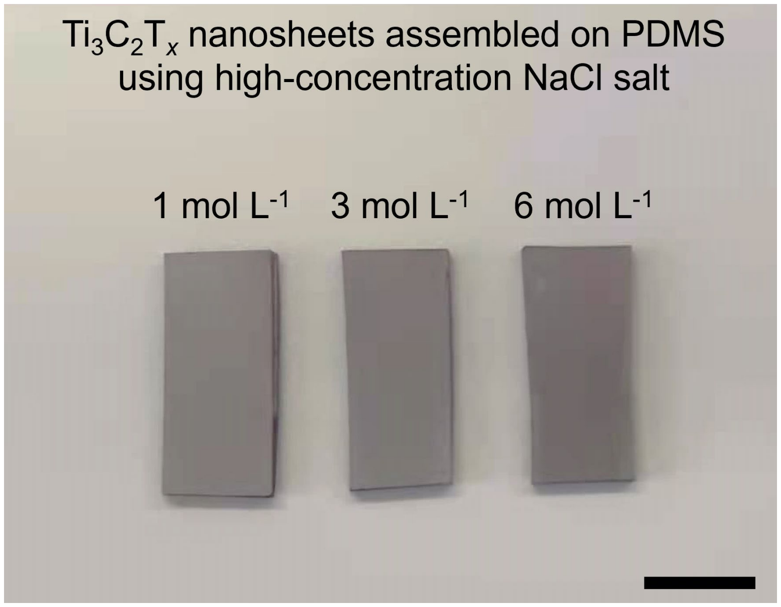

Supplementary Figure 4. Digital images of $Ti_3C_2T_x$ nanosheets assembled on PDMS using high-concentration NaCl salt. Scale bar, 4 mm.

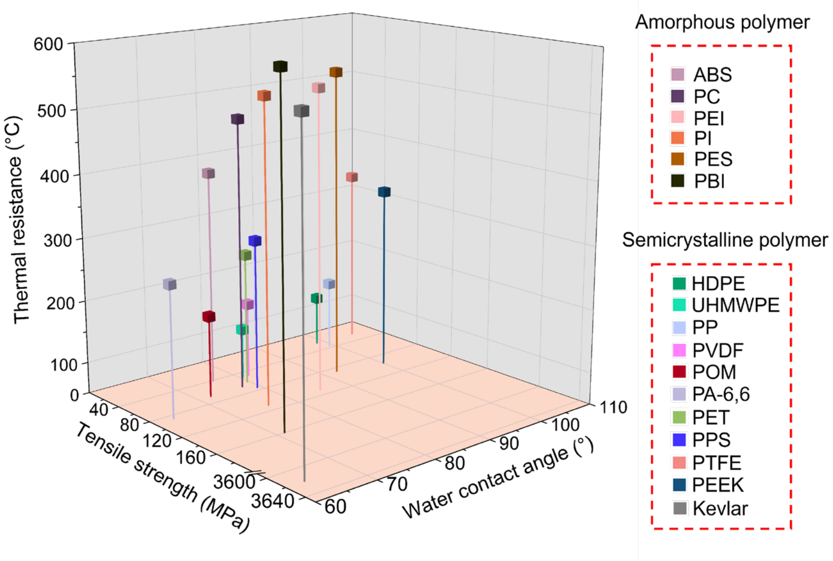

Supplementary Figure 5. High-performance polymer collection and the corresponding tensile strength, thermal resistance, and water contact angle[3,4]. The polymer includes amorphous polymer and semicrystalline polymer. The thermal resistance of amorphous polymers is defined as the initial degradation temperature and the thermal resistance of semicrystalline polymers is defined as the melting point.

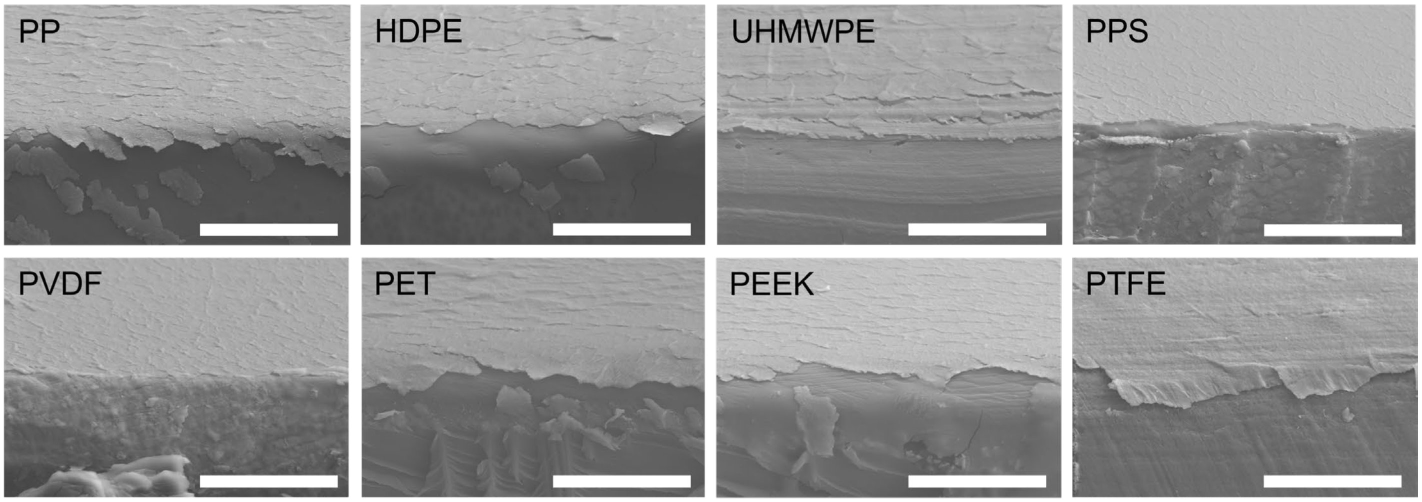

Supplementary Figure 6. Tilted-angle SEM images of cross-sections of Na-$Ti_3C_2T_x$ nanosheets assembled on diverse polymer films. Scale bars, 50 μm.

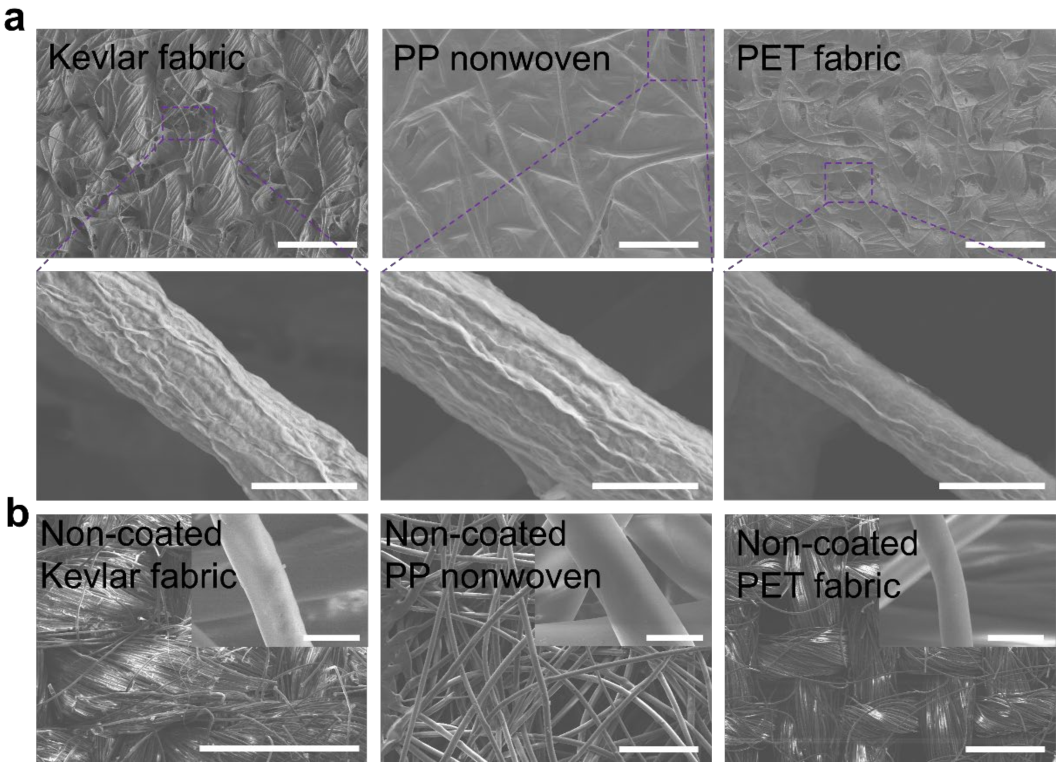

Supplementary Figure 7. SEM images of Na-$Ti_3C_2T_x$ nanosheets assembled on polymer fibers. a, Na-$Ti_3C_2T_x$ nanosheets on different polymer fibers. SEM images show that the Na-$Ti_3C_2T_x$ nanosheets not only wrap the surface of fibers but create bridges to connect the fibers. Also, the assembled Na-$Ti_3C_2T_x$ nanosheets on a single polymer fiber feature a wrinkled structure. Scale bars for top images, 1 mm. Scale bars for bottom images, 20 μm. b, non-coated polymer fibers. Scale bars for zoom-out images, 1 mm. Scale bars for insets, 20 μm.

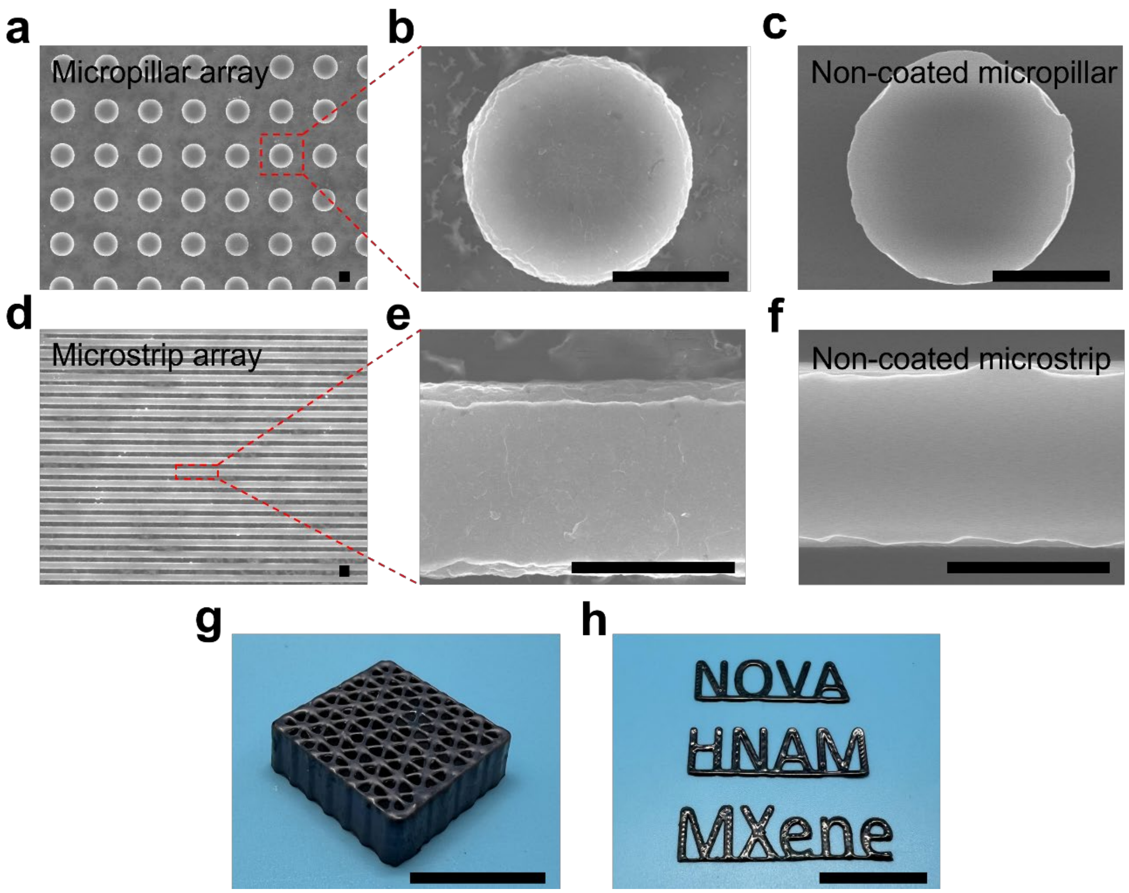

Supplementary Figure 8. Assembly of Na-$Ti_3C_2T_x$ nanosheets on micro-patterned and 3D printed PDMS substrates. a, b, SEM images of Na-$Ti_3C_2T_x$ nanosheets assembled on PDMS substrates with micropillar array. c, Non-coated micropillar. d, e, SEM images of Na-$Ti_3C_2T_x$ nanosheets assembled on PDMS substrates with microstrip array. f, Non-coated microstrip. g, h, SEM images of Na-$Ti_3C_2T_x$ nanosheets assembled on 3D printed PDMS substrates. Scale bars for a-d, 10 μm. Scale bars for e-f, 5 μm. Scales bars for g-h, 1 cm.

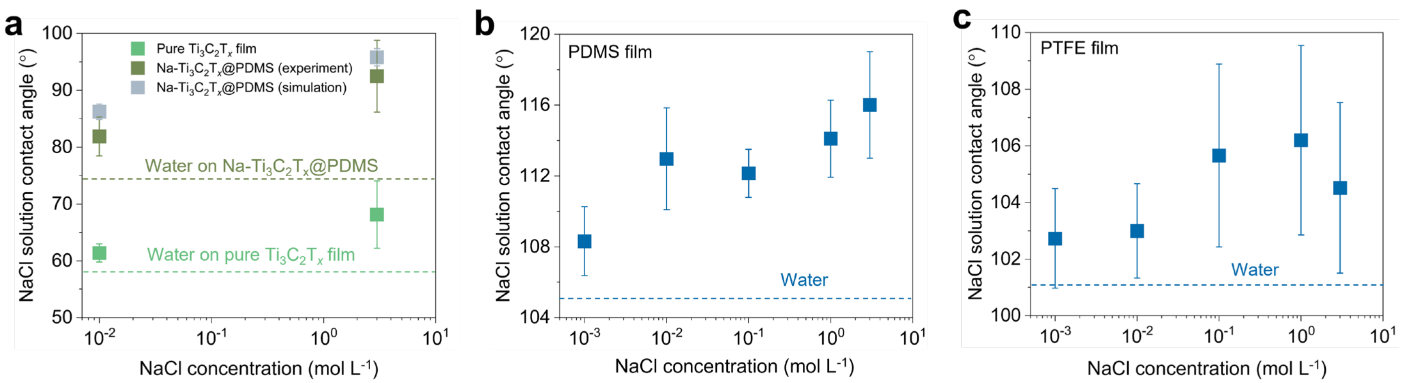

Supplementary Figure 9. Pure water and NaCl solution contact angle measurement on MXene and different polymer substrates. a, Pristine $Ti_3C_2T_x$ film obtained by vacuum filtration and on Na-$Ti_3C_2T_x$@PDMS obtained

by SAA. b, Pristine PDMS film. c, Pristine PTFE film. Because the concentration axes are plotted in logarithm scale, the dashed lines represent the pure water contact angles on the different substrates. All error bars are based on the standard deviation of 5 samples. Overall, with the increase of salt concentration (pure water, 0.01 mol L^{-1} NaCl, and 3 mol L^{-1} NaCl), we have identified a trend of increased contact angle for MXene film, PDMS substrate, and PTFE substrates. As shown in Supplementary Fig S9a, the higher contact angle of Na-$Ti_3C_2T_x$ compared to pristine $Ti_3C_2T_x$ suggests two important functions of salt in the assembly process. First, NaCl in the water will increase the contact angles of water for both pristine MXene film and polymer substrate. Second, the metal ions that adhere to the surface of MXene also change the surface properties of MXene and make it more hydrophobic. Both effects lead to the energetically favorable assembly of MXene on polymer.

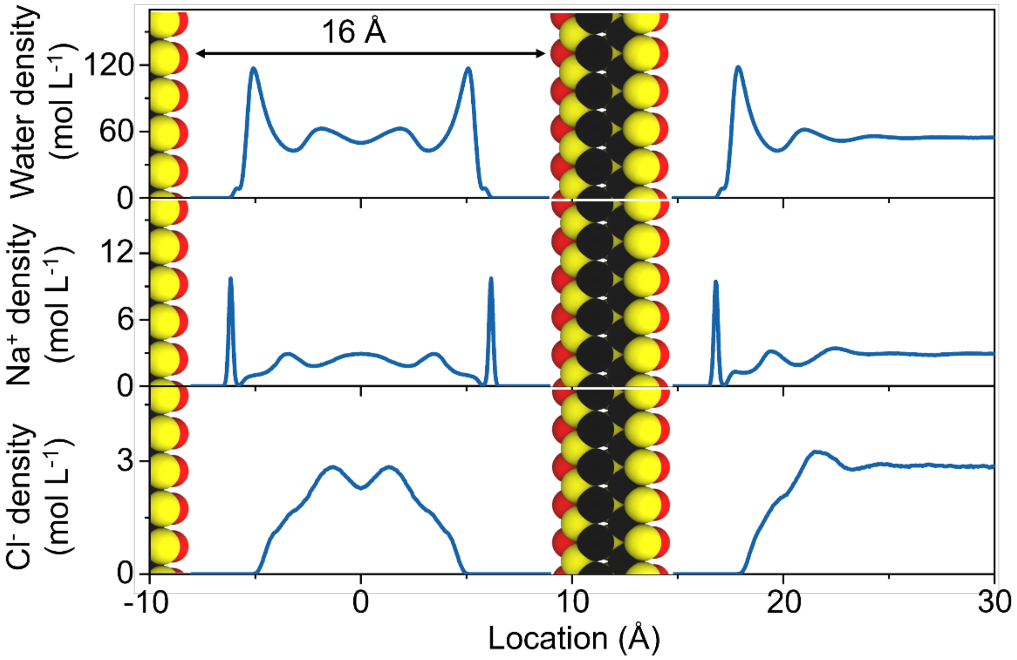

Supplementary Figure 10. Electric double layer (EDL) simulation on the surface of $Ti_3C_2T_x$ nanosheets. The results show the inner and outer molar densities of water molecules (top), Na^+ ions (middle), and Cl^- ions (bottom) of $Ti_3C_2T_x$ nanosheets when the distance of two $Ti_3C_2T_x$ nanosheets is 16 Å.

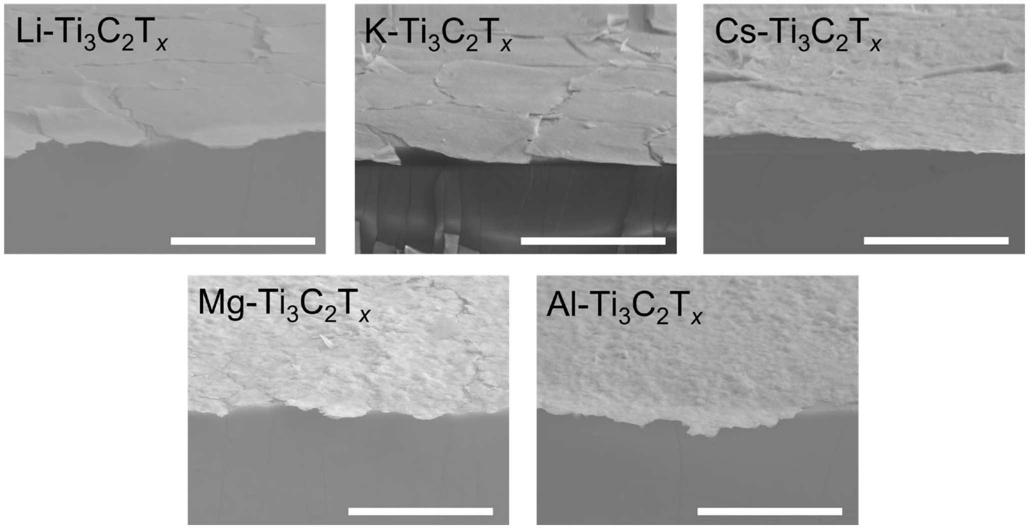

Supplementary Figure 11. SEM images of tilted angle view of salt-treated $Ti_3C_2T_x$ nanosheets assembled on PDMS. Scale bars, 50 µm. The samples here undergo dip-coating assembly for 15 min.

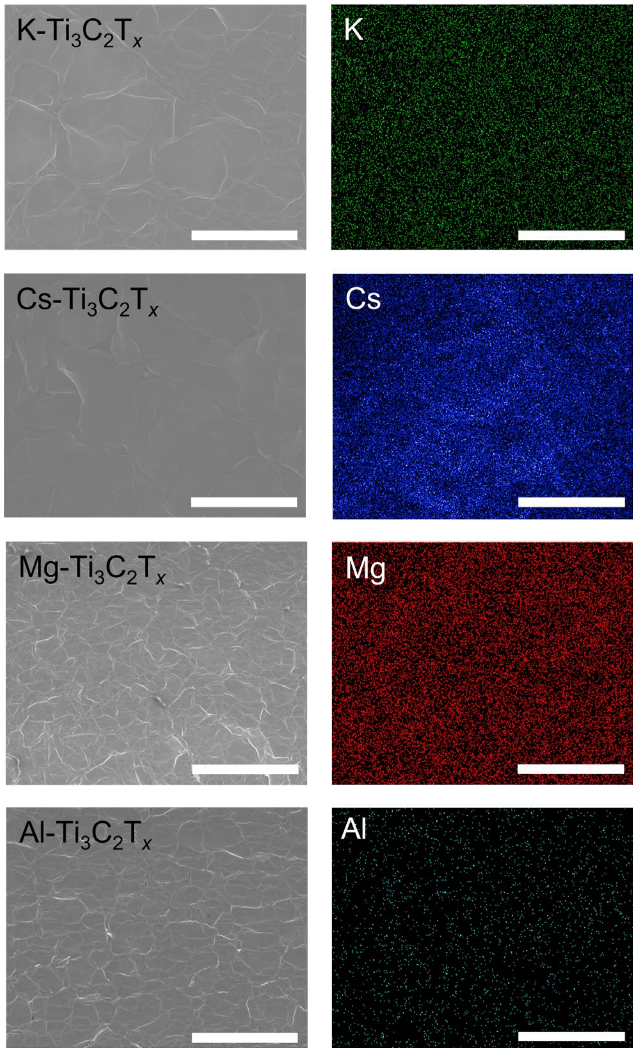

Supplementary Figure 12. SEM images of the top surface of salt-treated $Ti_3C_2T_x$ nanosheets assembled on PDMS and corresponding EDS mapping. Scale bars, 5 μm. The samples here undergo dip-coating assembly for 15 min.

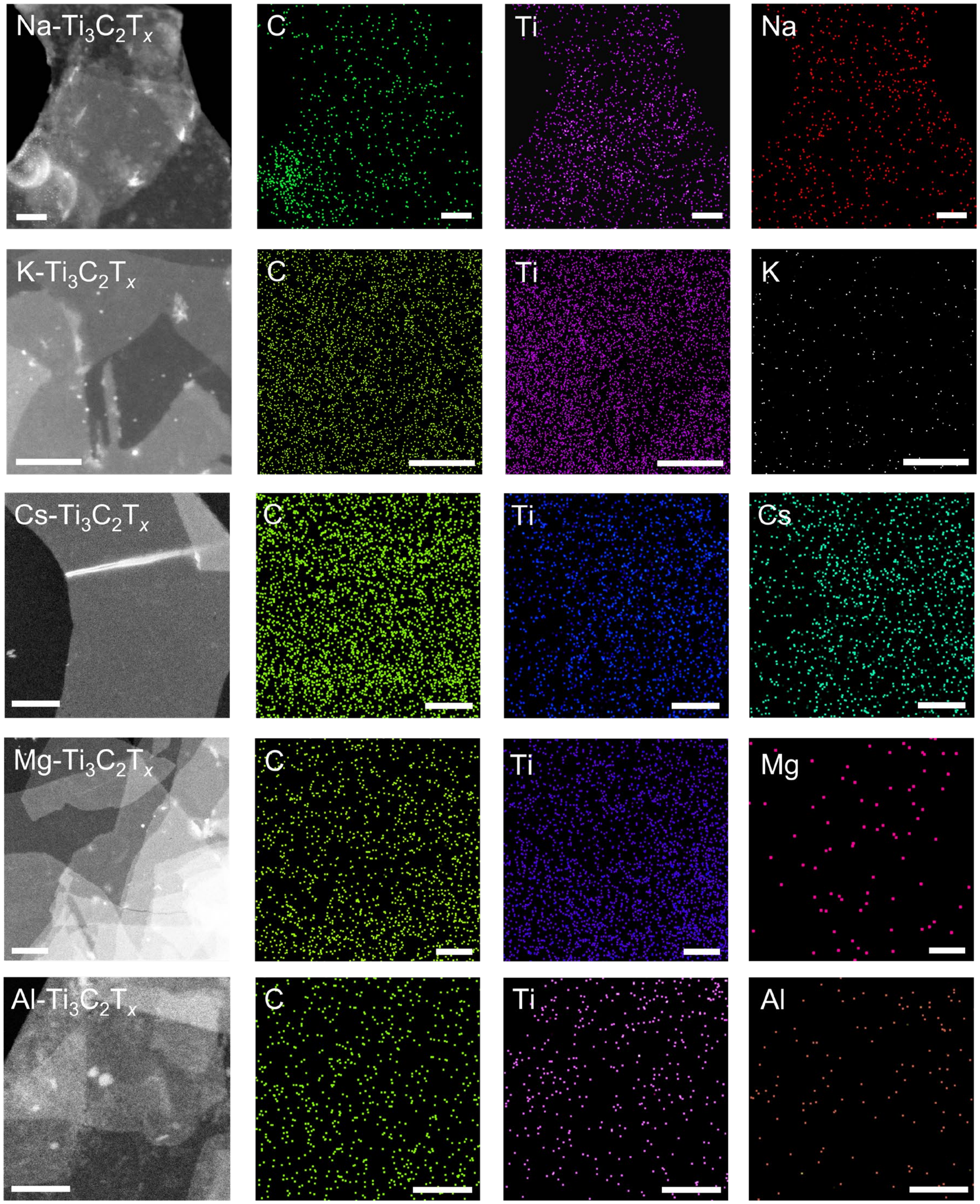

Supplementary Figure 13. HAADF images of different salt-treated $Ti_3C_2T_x$ nanosheets and corresponding EDS maps. Scale bars, 100 nm.

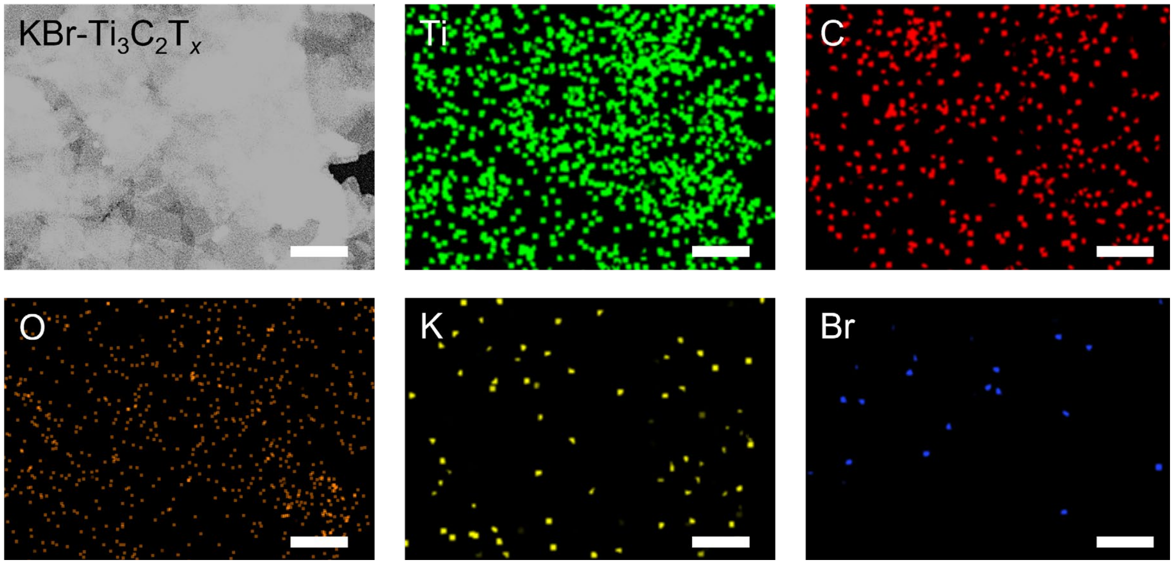

Supplementary Figure 14. HAADF images of KBr salt (concentration: 0.01 mol L^{-1}) treated $Ti_3C_2T_x$ nanosheets and corresponding EDS mapping. Scale bars, 50 nm.

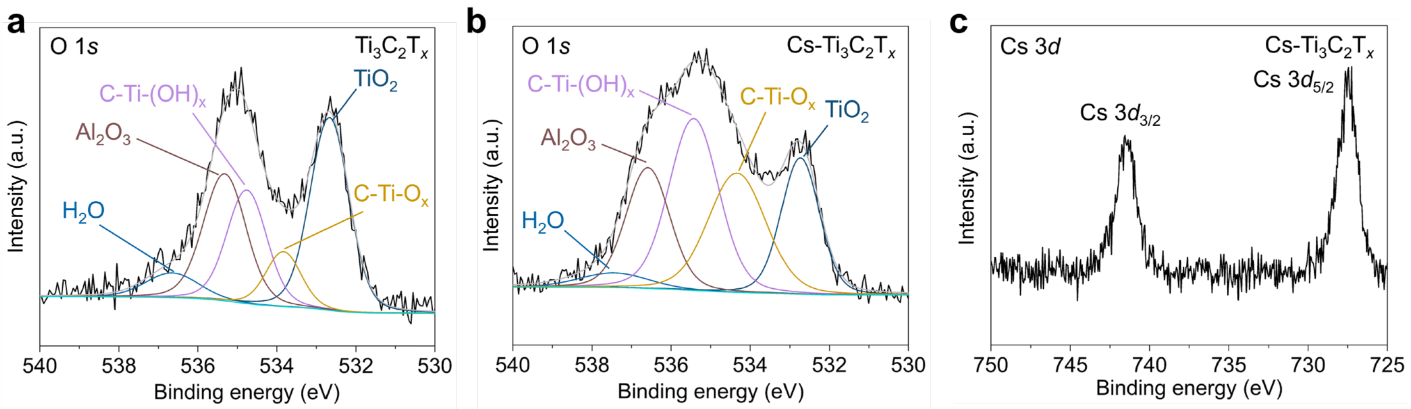

Supplementary Figure 15. XPS spectra of pristine $Ti_3C_2T_x$ and Cs-$Ti_3C_2T_x$ nanosheets. a, O 1s spectrum of $Ti_3C_2T_x$ nanosheets. b, O 1s spectrum of Cs-$Ti_3C_2T_x$ nanosheets. c, Cs 3d spectrum of Cs-$Ti_3C_2T_x$ nanosheets. Compared with $Ti_3C_2T_x$ nanosheets, the C-Ti-O_x and C-Ti-$(OH)_x$ peaks in O 1s spectra of Cs-$Ti_3C_2T_x$ nanosheets shift to higher binding energy, which indicates the Cs ion attachment on $Ti_3C_2T_x$ nanosheet surface with O-based groups[5].

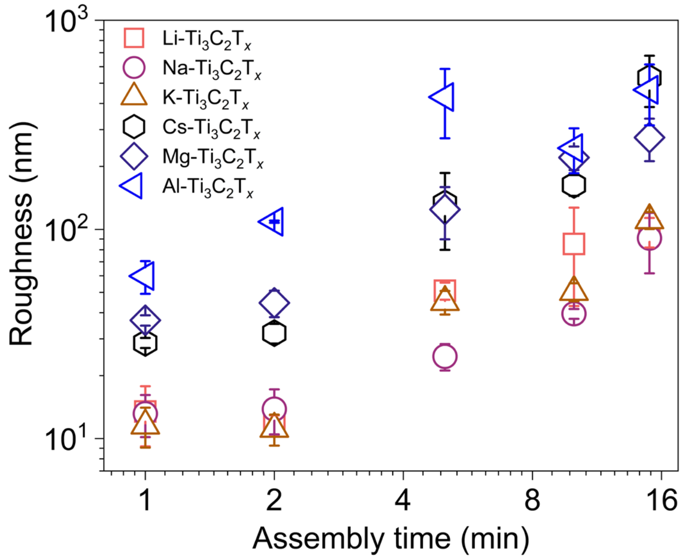

Supplementary Figure 16. Roughness evolution versus assembly time of salt-treated $Ti_3C_2T_x$ nanosheet assemblies on PDMS substrates. The error bars are based on the standard deviation of 3 samples.

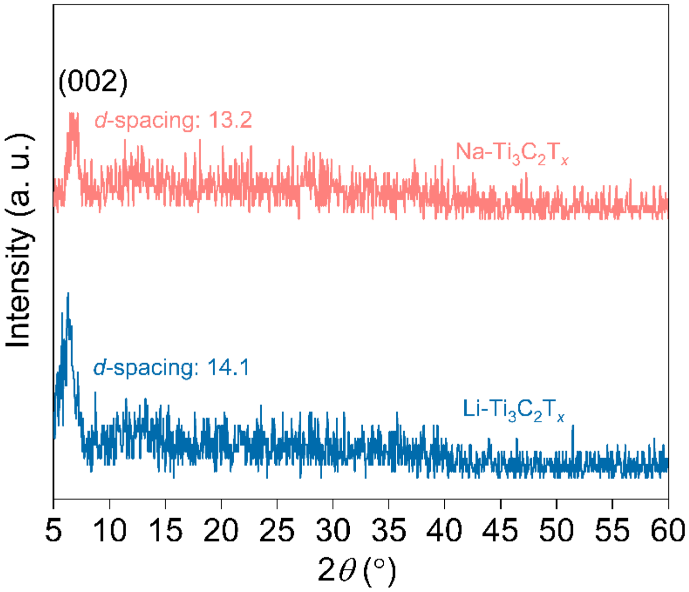

Supplementary Figure 17. XRD spectra of salt-treated $Ti_3C_2T_x$ nanosheet assemblies on PDMS. As salt is added to $Ti_3C_2T_x$ aqueous suspension, the cations will be intercalated into $Ti_3C_2T_x$ layers (i.e., ion exchange)[6]. Thus, compared with pristine $Ti_3C_2T_x$ nanosheets (Supplementary Fig. 1a), the (002) planes of Li-$Ti_3C_2T_x$ and Na-$Ti_3C_2T_x$ shift to the location with a lower 2θ value. Besides, the (002) plane peak intensities greatly weakened, which may be ascribed to the disordered $Ti_3C_2T_x$ structure after intercalation[7]. The *d*-spacing along the (002) plane of Li-$Ti_3C_2T_x$ (14.1 Å) is larger than Na-$Ti_3C_2T_x$ (13.2 Å), which is due to the larger solvated Li^+ cation than Na^+ cation.

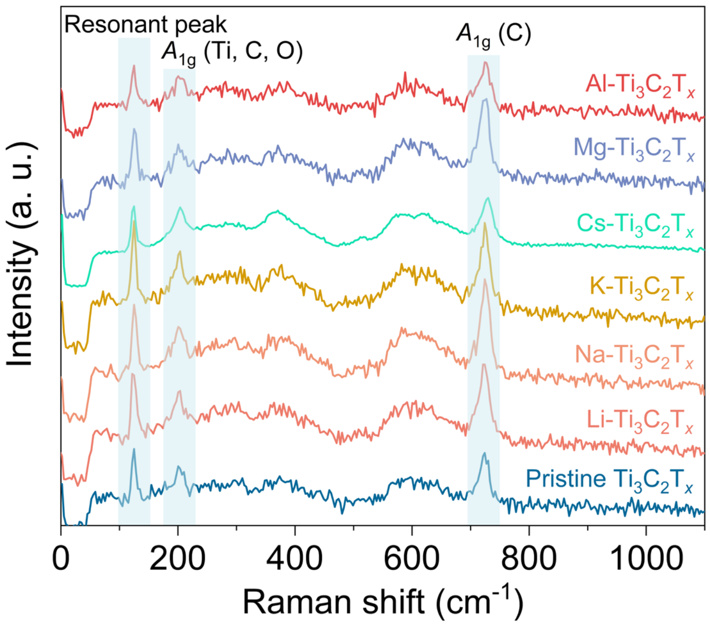

Supplementary Figure 18. Raman spectra of salt-treated $Ti_3C_2T_x$ nanosheets. There are three distinct peaks existing for the pristine $Ti_3C_2T_x$ and cation-$Ti_3C_2T_x$ on PDMS: resonant peak at around 124 cm^{-1}, A_{1g} (Ti, C, O) at around 200 cm^{-1}, and A_{1g} (C) at around 724 cm^{-1}. Comparing pristine $Ti_3C_2T_x$ with cation-$Ti_3C_2T_x$, A_{1g} out-of-plane vibration modes at 200 cm^{-1} and 724 cm^{-1} for C and Ti atoms experience few changes, indicating stable structural and electronic properties. Also, TiO_2 signals were not detected from salt-treated $Ti_3C_2T_x$ nanosheets, which demonstrates high resistance to hydrolysis and oxidation[8,9]. Note that we used a 785 nm laser to excite the $Ti_3C_2T_x$ with a ×20 objective.

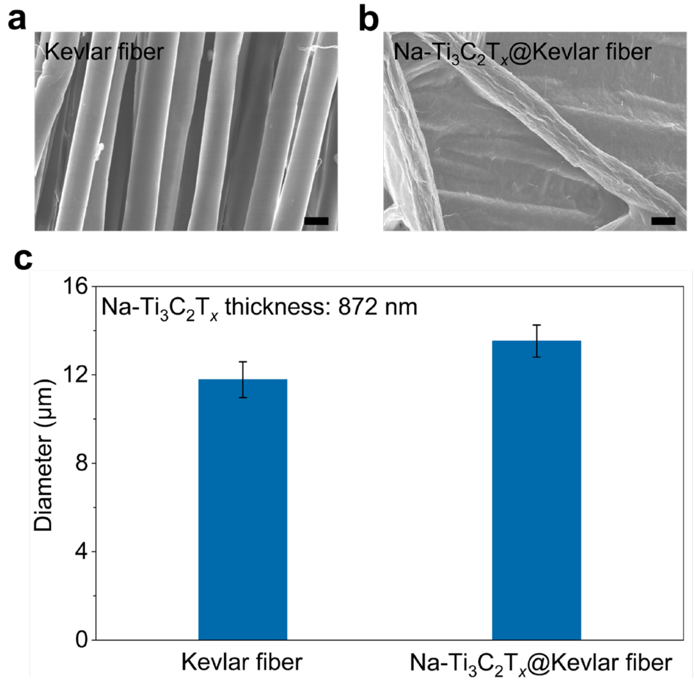

Supplementary Figure 19. Na-$Ti_3C_2T_x$ nanosheet assembly thickness on single Kevlar fiber. a, Representative SEM image of Kevlar fiber. b, Representative SEM image of Na-$Ti_3C_2T_x$@Kevlar. Scale bars, 10 μm. c, Diameter summary of Kevlar fiber and Na-$Ti_3C_2T_x$@Kevlar and calculated Na-$Ti_3C_2T_x$ thickness. The calculated diameters

are based on the average values for 30 fibers. The error bars are based on the standard deviation of diameters for 30 fibers. It should be noted that the average thickness may include the extended wrinkles, which are thicker than the rest of the film.

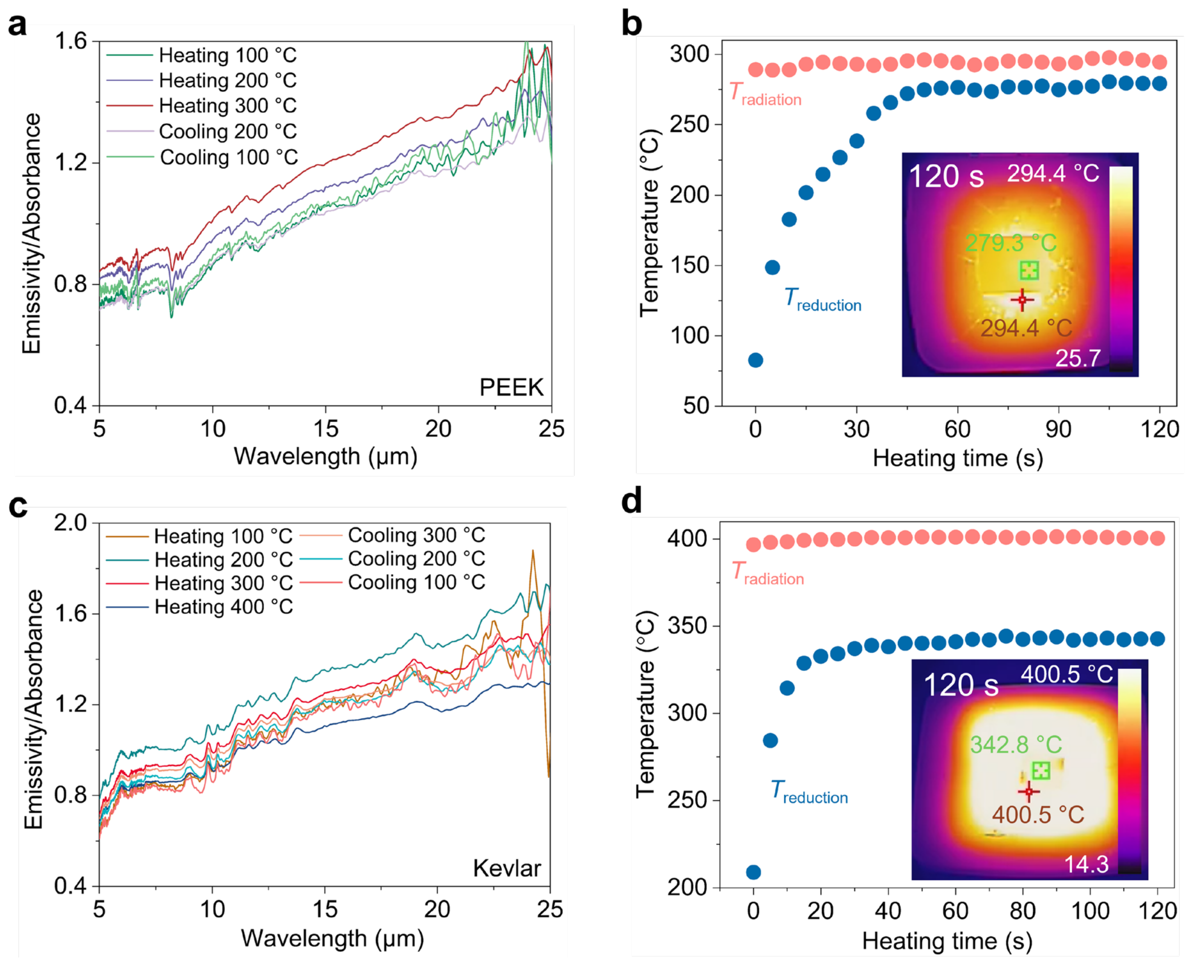

Supplementary Figure 20. Thermal emissivity/absorbance and thermal camouflage performance of pure polymer substrates. a, Thermal emissivity/absorbance of PEEK film at different temperatures during one heating-cooling cycle. b, Thermal camouflage performance of PEEK film up to 300 °C. c, Thermal emissivity/absorbance of Kevlar fabric at different temperatures during one heating-cooling cycle. d, Thermal camouflage performance of Kevlar fabric up to 400 °C. For both polymers, the temperature difference $T_{radiation}$ -$T_{reduction}$ at the highest $T_{radiation}$ is much smaller compared to the polymer coated with Na-$Ti_3C_2T_x$. Therefore, the thermal camouflage performance of Na-$Ti_3C_2T_x$ coated polymer can be mainly attributed to Na-$Ti_3C_2T_x$ coating. Note that the edges of pure Kevlar fabric appear at lower temperatures than the center regions. The reason is that the Kevlar fabric edges start to deform and detach from the hot plate under high temperatures.

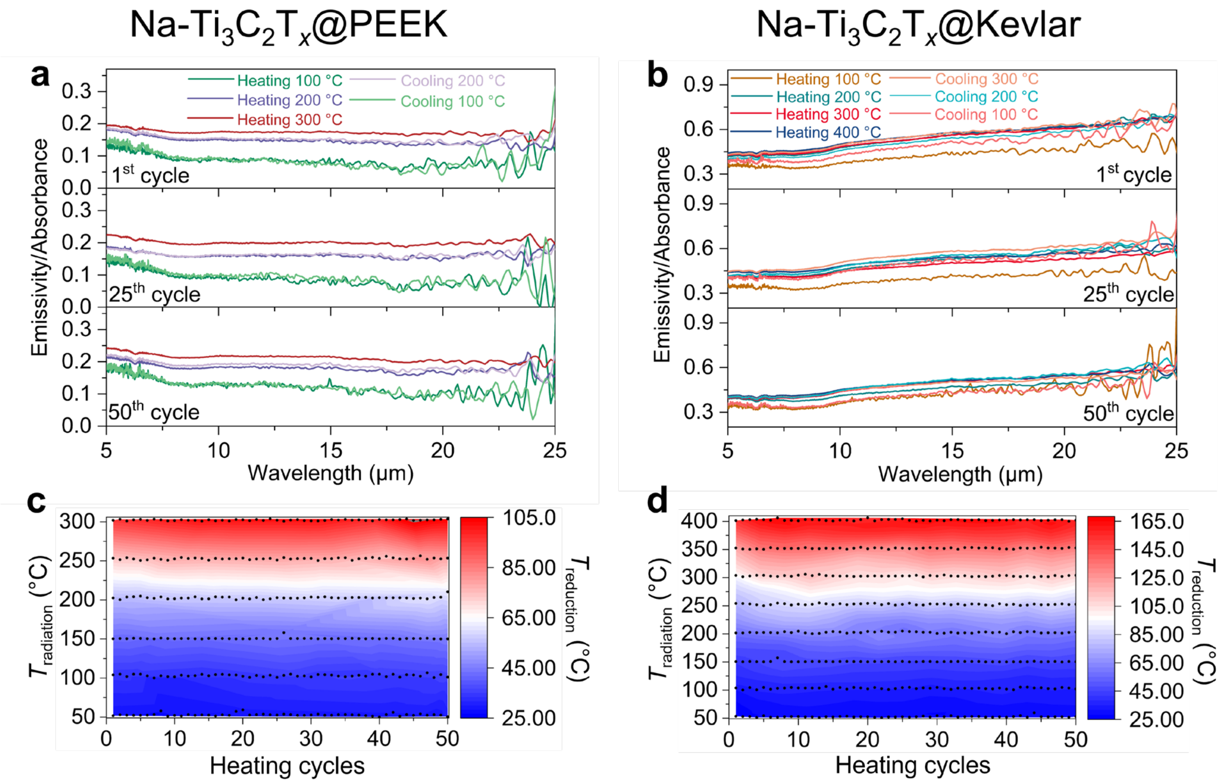

Supplementary Figure 21. Thermal camouflage performance of Na-$Ti_3C_2T_x$@PEEK and Na-$Ti_3C_2T_x$@Kevlar at high temperatures. a, The emissivity/absorbance of (a) Na-$Ti_3C_2T_x$@PEEK and (b) Na-$Ti_3C_2T_x$@Kevlar during heating-cooling cycles. c, d, Radiation ($T_{radiation}$) and reduction ($T_{reduction}$) temperature evolution (c) Na-$Ti_3C_2T_x$@PEEK and (d) Na-$Ti_3C_2T_x$@Kevlar during 50 heating cycles.

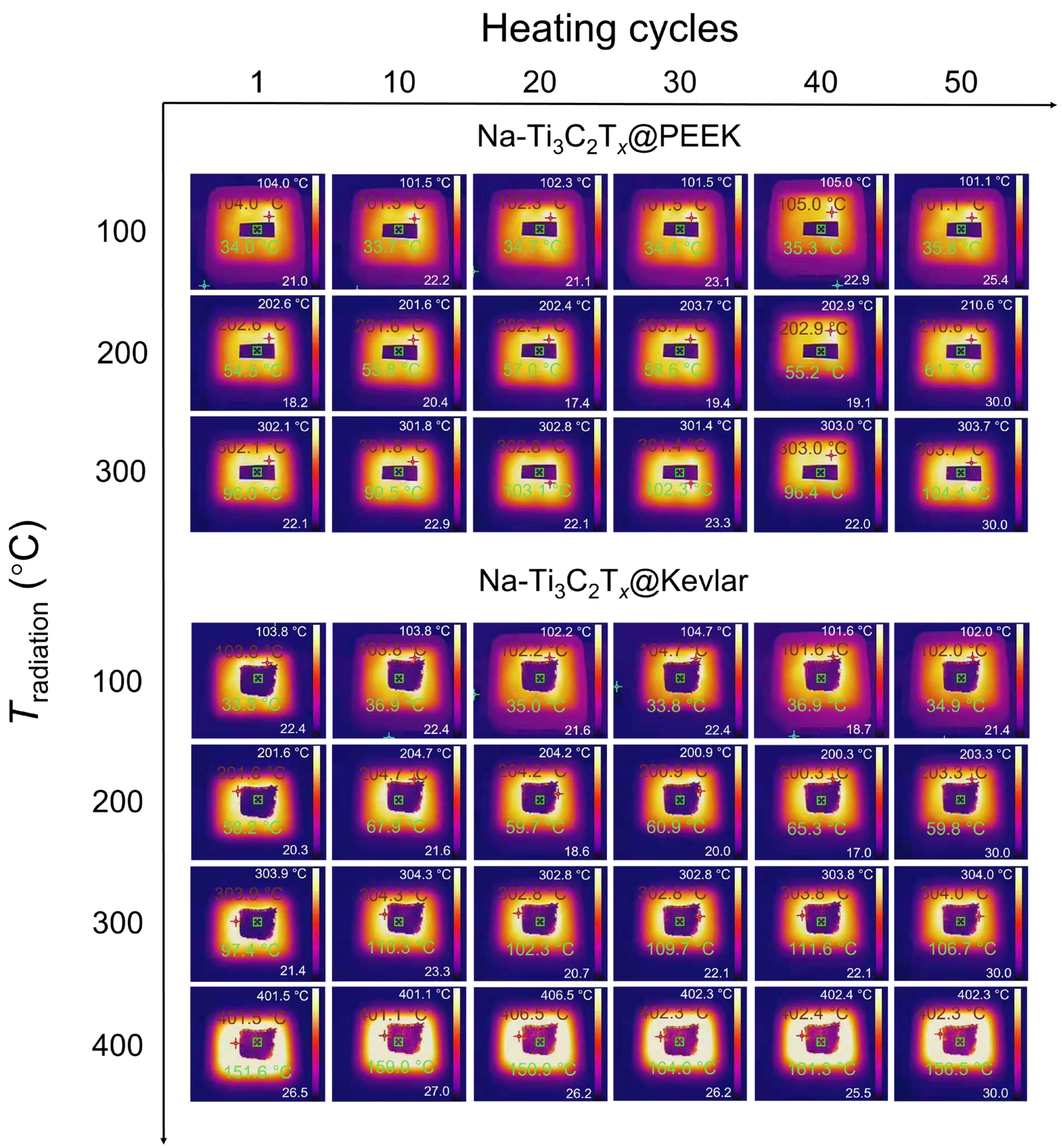

Supplementary Figure 22. IR images of Na-$Ti_3C_2T_x$@PEEK and Na-$Ti_3C_2T_x$@Kevlar during heating cycles.

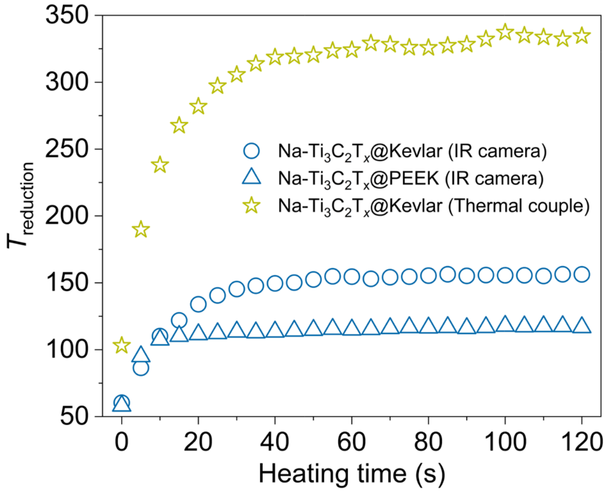

Supplementary Figure 23. Reduction temperature ($T_{reduction}$) evolution of Na-$Ti_3C_2T_x$@PEEK at 300 °C and Na-$Ti_3C_2T_x$@Kevlar at 400 °C for 120 s. Through this $T_{reduction}$ evolution determination at the initial stage, we have concluded that after the first 120 seconds, the $T_{reduction}$ will stabilize without increasing or decreasing. Thus, for the long-term thermal camouflage performance, we started to record the data after 120 s. The real temperature on Na-$Ti_3C_2T_x$@Kevlar was also tested by a thermal couple under ~400 °C, appearing to have a much higher value than the one measured by the IR camera.

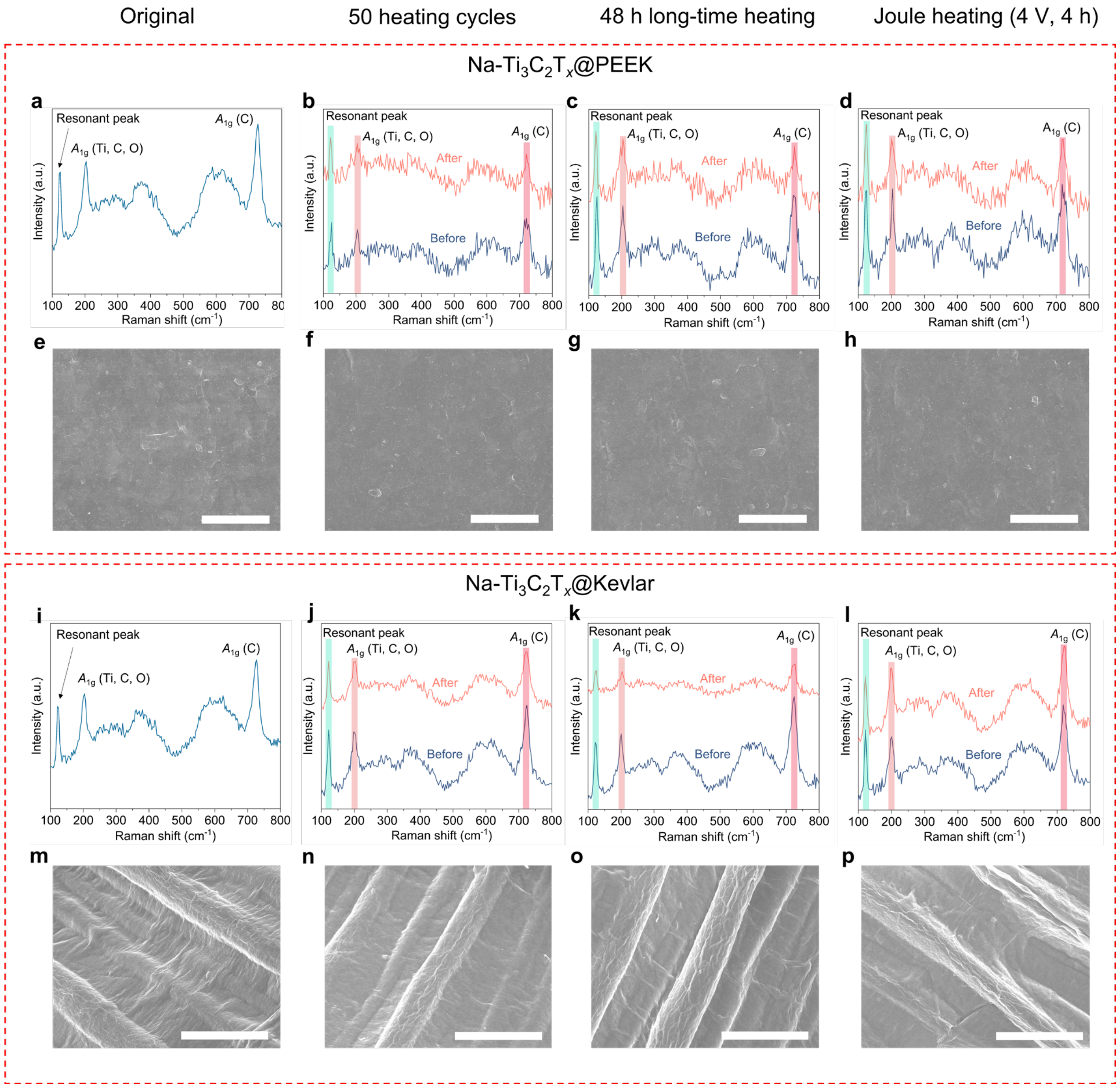

Supplementary Figure 24. SEM and Raman characterization of Na-$Ti_3C_2T_x$@PEEK and Na-$Ti_3C_2T_x$@Kevlar before and after thermal camouflage and joule heating test. a, e, Raman spectrum and SEM image of original Na-$Ti_3C_2T_x$@PEEK. b-d, Raman spectra of Na-$Ti_3C_2T_x$@PEEK before and after 50 heating cycles, 48 h long-time heating, and joule heating test (4 V, 4 h). f-i, SEM images of Na-$Ti_3C_2T_x$@PEEK after 50 heating cycles, 48 h long-time heating, and joule heating test (4 V, 4 h). i, m, Raman spectrum and SEM image of original Na-$Ti_3C_2T_x$@Kevlar. j-l, Raman spectra of Na-$Ti_3C_2T_x$@Kevlar before and after 50 heating cycles, 48 h long-time heating, and joule heating test (4 V, 4 h). n-p, SEM images of Na-$Ti_3C_2T_x$@Kevlar after 50 heating cycles, 48 h long-time heating, and joule heating test (4 V, 4 h). Scale bars for e-h, 5 µm. Scale bars for m-p, 50 µm. Compared with the Raman spectra before and after joule heating and thermal camouflage testing, we can see that Raman peaks of the Na-$Ti_3C_2T_x$ retain similar positions, even though there is a small decrease in intensity, indicative of minimal structure damage.

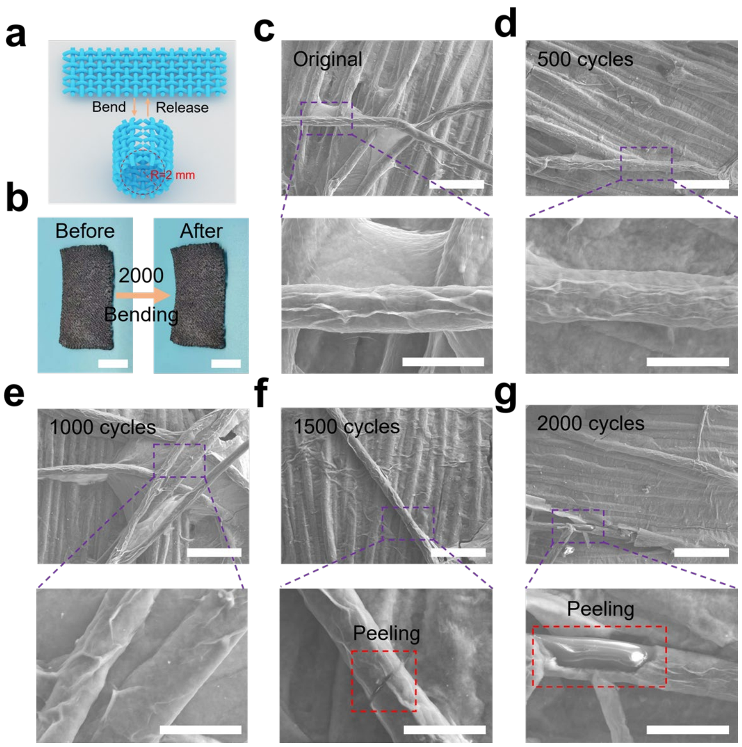

Supplementary Figure 25. Morphology characterization of Na-$Ti_3C_2T_x$@Kevlar during bending cycles. a, Schematic of bending test. b, Digital images of Na-$Ti_3C_2T_x$@Kevlar before and after 2000 bending cycles. Scale bars, 4 mm. c-g, SEM images of Na-$Ti_3C_2T_x$@Kevlar within 2000 bending cycles. Scale bars for low (top) and high (bottom) magnification are 100 µm and 30 µm, respectively.

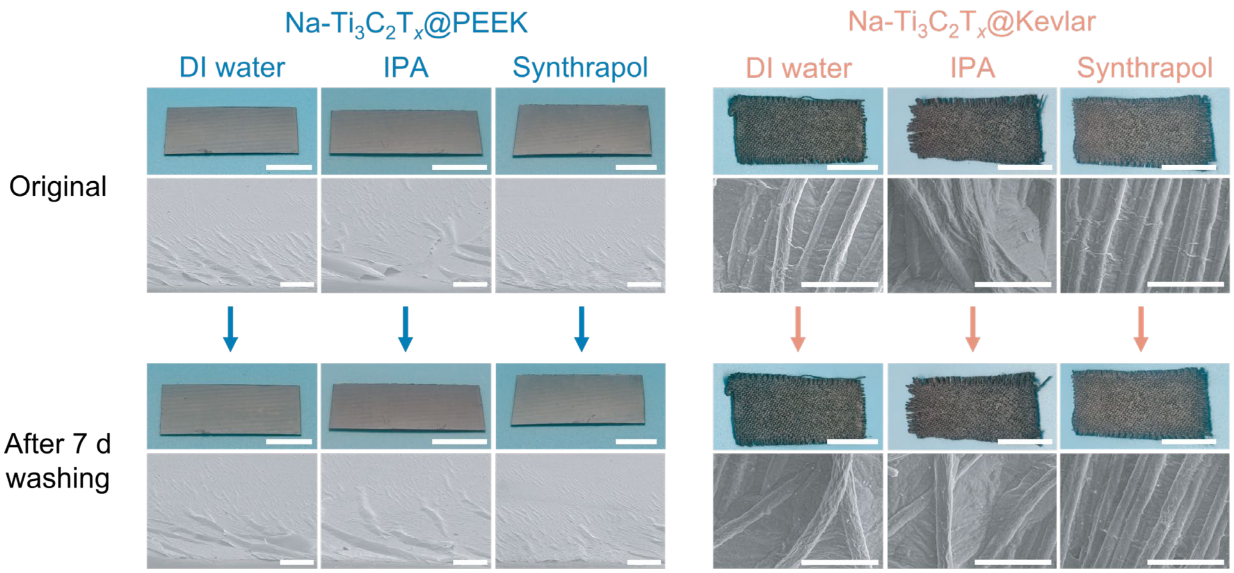

Supplementary Figure 26. Characterization of Na-$Ti_3C_2T_x$@PEEK and Na-$Ti_3C_2T_x$@Kevlar before and after long-term washing using diverse solutions. Digital and SEM images of Na-$Ti_3C_2T_x$@PEEK and Na-$Ti_3C_2T_x$@Kevlar before and after washing for 7 days using DI water, IPA, and Synthrapol solution (10 vol%).

Scale bars for digital images, 10 mm. Scale bars for SEM images, 100 μm. Digital and SEM images show no obvious coating damage or defects from both macroscopic and microscopic perspectives. Thus, we can conclude that the Na-$Ti_3C_2T_x$ coating can retain its structural integrity during the long-term washing process.

Supplementary Table 1. Information summary of materials used in this work.

Materials	Supplier	Remark
Ti_3AlC_2 MAX phase	Materials Research Center, Ukraine	<40 µm particle size
HF	Acros Organics	29 M (49 wt. %)
HCl	Fisher Scientific	12M
LiCl	Sigma-Aldrich	≥99.0%, for molecular biology
NaCl	VWR Chemicals BDH	≥99.0%, ACS
$MgCl_2$	VWR Life Science	98%, high purity, anhydrous
$AlCl_3$	BeanTown Chemical	100%, anhydrous
KCl	VWR Chemicals BDH	99.0-100.5%, ACS
$CaCl_2$	Fisher BioReagents	≥99.0%, dihydrate
$ScCl_3$	Thermo Scientific Chemicals	≥99.9%, anhydrous (REO, rare earth oxide basis)
$TiCl_4$	BeanTown Chemical	≥99.99% (trace metals basis)
VCl_3	Thermo Scientific Chemicals	97%
$CrCl_3$	Thermo Scientific Chemicals	≥99.9%, anhydrous (metals basis)
$MnCl_2$	Thermo Scientific Chemicals	97%
$FeCl_3$	Sigma-Aldrich	97%, ACS, hexahydrate
$CoCl_2$	Aldon Corp SE	100%, hexahydrate
$NiCl_2$	Aldon Corp SE	100%, hexahydrate
$CuCl_2$	Aldon Corp SE	>98%, dihydrate
$ZnCl_2$	Sigma-Aldrich	≥98.0%, anhydrous
$GaCl_3$	BeanTown Chemical	≥99.999%, anhydrous (trace metals basis)
$GeCl_4$	Thermo Scientific Chemicals	≥99.9999%, anhydrous (metals basis)
RbCl	BeanTown Chemical	≥99.5%, anhydrous (trace metals basis)
$SrCl_2$	Aldon Corp SE	100%, hexahydrate
YCl_3	Thermo Scientific Chemicals	≥99.9%, hydrate (REO, rare earth oxide basis), REacton®
$ZrCl_4$	Strem Chemicals Inc MS	≥99.5%, anhydrous
$NbCl_5$	Thermo Scientific Chemicals	99.8%, anhydrous
$MoCl_3$	BeanTown Chemical	≥99.5%, anhydrous (trace metals basis)
$RuCl_3$	BeanTown Chemical	≥99.9%, hydrate (trace metals basis)
$RhCl_3$	BeanTown Chemical	≥99.9%, anhydrous (trace metals basis)
$PbCl_2$	BeanTown Chemical	≥99.9%, anhydrous (trace metals basis)
$CdCl_2$	Thermo Scientific Chemicals	≥99.0 %, anhydrous, ACS
$InCl_3$	Thermo Scientific Chemicals	≥99.999%, anhydrous (metals basis)
$SnCl_4$	Thermo Scientific Chemicals	≥98.0 %, pentahydrate, extra pure
$SbCl_3$	BeanTown Chemical	≥99.9%, anhydrous (trace metals basis)
CsCl	VWR Life Science	≥99.9%, anhydrous, ultra-pure grade
$BaCl_2$	Aldon Corp SE	100%, Dihydrate
$LaCl_3$	SPECTRUM CHEMICAL MFG CORP	64.5-70.0%, hydrate
$HfCl_4$	Thermo Scientific Chemicals	≥98%, anhydrous (metals basis excluding Zr) (max. 2.7% Zr)
WCl_6	Thermo Scientific Chemicals	99%, anhydrous
$ReCl_3$	Thermo Scientific Chemicals	100%, anhydrous
$OsCl_3$	BeanTown Chemical	≥99.99%, trihydrate (trace metals basis)
$IrCl_3$	TCI America	Hydrate
$PtCl_4$	Thermo Scientific Chemicals	99%, anhydrous
$AuCl_3$	BeanTown Chemical	Anhydrous
$BiCl_3$	Thermo Scientific Chemicals	≥99.997%, anhydrous (metals basis), ultra dry
NaF	BeanTown Chemical	≥99%, anhydrous
NaBr	BeanTown Chemical	≥99%, anhydrous
NaI	Thermo Scientific Chemicals	≥99%, anhydrous
$NaNO_3$	Sigma-Aldrich	≥99.0%, ACS, anhydrous
$NaC_6H_7O_6$	BeanTown Chemical	100%, anhydrous
$AgNO_3$	BeanTown Chemical	99.9%, anhydrous (trace metals basis)
KBr	BeanTown Chemical	≥99%, anhydrous
PDMS	Dow Corning Corp	SYLGARD™ 184
PP	CS Hyde Company	Thickness: 0.254 mm
HDPE film	CS Hyde Company	Thickness: 0.381 mm
UHMWPE film	CS Hyde Company	Thickness: 0.254 mm
PPS film	CS Hyde Company	Thickness: 0.254 mm
PVDF film	CS Hyde Company	Thickness: 0.254 mm
PTFE film	CS Hyde Company	Thickness: 0.254 mm
PET film	Benecreat	Thickness: 0.2 mm
PEEK film	CS Hyde Company	Thickness: 0.381 mm
PES film	CS Hyde Company	Thickness:0.0762 mm
ABS film	CS Hyde Company	Thickness: 0.254 mm
PI film	CS Hyde Company	Thickness: 0.254 mm
PEI film	CS Hyde Company	Thickness: 0.254 mm
PBI film	PBI Performance Products, Inc.	Thickness: 0.055 mm
POM film	CS Hyde Company	Thickness: 0.254 mm
Nylon-6,6 film	CS Hyde Company	Thickness: 0.254 mm
PC film	Zonon	Thickness: 0.508 mm
PP nonwoven	BYD Care	Thickness: 0.1 mm
PET fabric	Testfabrics, Inc.	Thickness: 0.2 mm
Kevlar fabric	CS Hyde Company	Thickness: 0.6096 mm, square yard

Supplementary Table 2. Summary of reported freestanding $Ti_3C_2T_x$ MXene film and $Ti_3C_2T_x$ coatings on polymers.

Building block	Substrate	Substrate modification	Assembly method	Assembly thickness (μm)	Conductivity (S cm^{-1})	Ref.
$Ti_3C_2T_x$	Freestanding	-	Vacuum filtration	~3.6	~25000	10
$Ti_3C_2T_x$	Freestanding	-	Blade coating	0.214	15100	11
$Ti_3C_2T_x$	Freestanding	-	Vacuum filtration	3.3	2402	12
$Ti_3C_2T_x$	Freestanding	-	Vacuum filtration	6	10400	13
$Ti_3C_2T_x$	Freestanding	-	Drop casting	23.2	7000	14
$Ti_3C_2T_x$	Freestanding	-	Interfacial assembly	0.010	3226	15
$Ti_3C_2T_x$	Freestanding	-	Blade coating	3.6	9855	16
$Ti_3C_2T_x$/GO	Freestanding	-	Vacuum filtration	7	461.7	17
$Ti_3C_2T_x$/CNF	Freestanding	-	Vacuum filtration	47	7	18
$Ti_3C_2T_x$/PDA	Freestanding	-	Vacuum filtration	6.95	5141	19
$Ti_3C_2T_x$/CNF	Freestanding	-	Vacuum filtration	6	1120	20
$Ti_3C_2T_x$/ANF	Freestanding	-	Vacuum filtration	37	931	21
$Ti_3C_2T_x$-Al^{3+}	Freestanding	-	Vacuum filtration	5	2656	22
$Ti_3C_2T_x$	PET film	Oxygen plasma	Spray coating	1.4	9276	23
S, N-$Ti_3C_2T_x$/GO	PET film	-	Blade coating	2.6	1198	24
$Ti_3C_2T_x$	PET film	Oxygen plasma	Spin casting	0.088	9880	25
$Ti_3C_2T_x$	PET film	Oxygen plasma	Spray coating	5.5	15000	26
**Na-$Ti_3C_2T_x$**	**PDMS film**	**-**	**Dip coating**	**0.132**	**20500**	**This work**

Supplementary Table 3. $Ti_3C_2T_x$ MXene assembled on different polymer substrates.

Material	Substrate	Substrate treatment	Assembly method	Assembly mechanism	Ref.
$Ti_3C_2T_x$	UHMWPE fiber	Plasma and bovine serum albumin treatment	Immersing	Electrostatic interaction	27
$Ti_3C_2T_x$	PVDF porous membrane	Hydrophilic as received	Vacuum filtration	Hydrogen bond interaction	28
$Ti_3C_2T_x$	PET and nylon fiber	O_2 plasma treatment and amino silanization	Dip coating	Hydrogen bond interaction	29
$Ti_3C_2T_x$	PC film	Air plasma treatment	Spray coating	Hydrogen bond interaction	30
$Ti_3C_2T_x$	Aramid fiber	Alkali and plasma treatment	Spray coating	Hydrogen bond interaction	31
$Ti_3C_2T_x$	PP fiber	O_2 plasma treatment and PEI grafting	Dip coating	Hydrogen bond interaction	32
$Ti_3C_2T_x$	PDMS film PET film Nylon fiber	Poly (diallyldimethylammonium chloride coating	Dip coating	Electrostatic interaction	33
Polydopamine-$Ti_3C_2T_x$	PEEK disc	Sulfonation	Immersing	Adhesive polymer bonding	34
$Ti_3C_2T_x$	Cellulose fiber	None	Dip coating	Hydrogen bond interaction	35
$Ti_3C_2T_x$	Cotton	None	Dip coating	Hydrogen bond interaction	36
$Ti_3C_2T_x$	PI foam	None	Dip coating	Hydrogen bond interaction	37

Supplementary Table 4. Roughness, sheet resistance, and thickness of Na-$Ti_3C_2T_x$ assemblies on various polymer substrates beyond PDMS. Note, the assembly time for each sample is fixed at 15 min, $Ti_3C_2T_x$ nanosheet and NaCl concentration are 5 mg mL^{-1} and 0.01 mol L^{-1}.

**Sample**	**Polymer and Na-$Ti_3C_2T_x$ roughness (nm)**	**Sheet resistance (Ohm sq^{-1})**	**Thickness (nm)**	**Conductivity (S cm^{-1})**
Na-$Ti_3C_2T_x$@PP	59±8, 88±65	3.9±0.3	147.0±59.3	17442.9
Na-$Ti_3C_2T_x$@HDPE	968.0±217, 661±65	4.4±0.5	257.2±124.8	8836.4
Na-$Ti_3C_2T_x$@UHMWPE	181±9, 244±43	4.0±0.8	196.0±193.4	12755.1
Na-$Ti_3C_2T_x$@PPS	29±3, 31±9	5.1±0.3	101.4±95.3	19337.1
Na-$Ti_3C_2T_x$@PVDF	46±2, 48±3	3.1±0.3	151.9±108.6	21236.4
Na-$Ti_3C_2T_x$@PTFE	144±23, 187±12	3.6±0.3	363.4±254.1	7643.9
Na-$Ti_3C_2T_x$@PET	44±4, 38±8	5.2±0.4	86.2±61.7	22309.5
Na-$Ti_3C_2T_x$@PEEK	57.0±5.0, 57±2	3.8±0.4	168.0 nm±49.3	15664.2
Na-$Ti_3C_2T_x$@PP nonvoven	-	5.0±1.2	-	-
Na-$Ti_3C_2T_x$@PET fabric	-	1.9±0.3	-	-
Na-$Ti_3C_2T_x$@Kevlar fabric	-	2.7±0.4	-	-

Supplementary Table 5. DLS results of $Ti_3C_2T_x$ nanosheet suspension.

		Pure $Ti_3C_2T_x$	$Ti_3C_2T_x$ with 0.01 M salt (no sonication)	$Ti_3C_2T_x$ with 0.01 M salt (15 min sonication)	$Ti_3C_2T_x$ with 0.01 M salt (15 min sonication and wait for 15 min)
Size (nm)	NaCl	1078±84	1265±98	1093±91	1089±176
	$AlCl_3$		15567±3673	1135±147	1169±261

We have compared DLS under three conditions: (1) pristine $Ti_3C_2T_x$ suspension (well-dispersed suspension without aggregation), (2) $Ti_3C_2T_x$ suspension after the addition of 0.01 M NaCl or $AlCl_3$ (the sonication was not turned on to redisperse the $Ti_3C_2T_x$ suspension), (3) redispersed $Ti_3C_2T_x$ suspension using sonication after the addition of 0.01 M NaCl or $AlCl_3$.

For Condition 1, the average size is 1078 nm. For Condition 2, the value increases to 1265 nm for NaCl and 15567±3673 for $AlCl_3$. Comparing Conditions 1 and 2 suggests that aggregation happens if we do not use sonication to redisperse the suspension.

For Condition 3, we used sonication to redisperse the $Ti_3C_2T_x$ suspension with 0.01 M salt for 15 min. This is exactly the process we performed before the assembly on the polymer substrates. We kept monitoring the suspension 's aggregation status after turning off the sonication for 15 min. The DLS results show an average size

of 1093 nm right after the sonication and 1089 nm 15 min later. The same situation applies to the $AlCl_3$-added suspension.

These results clarify the aggregation status of MXene nanosheets during the SAA process. First, adding salt without sonication will lead to aggregation. Second, sonication can redisperse MXene suspension and prevent aggregation. Third, after sonication redispersion, the colloidal suspension can remain dispersed during the assembly process, which takes up to 15 minutes for our samples.

Supplementary Table 6. Emissivity/absorbance of PEEK, Kevlar fabric, Na-$Ti_3C_2T_x$@PEEK, and Na-$Ti_3C_2T_x$@Kevlar during heating/cooling cycles.

Sample	Condition	Emissivity/absorbance (1st cycle)	Emissivity/absorbance (25th cycle)	Emissivity/absorbance (50th cycle)
Na-$Ti_3C_2T_x$@PEEK	Heating 100 °C	0.10	0.10	0.14
	Heating 200 °C	0.16	0.17	0.19
	Heating 300 °C	0.18	0.21	0.22
	Cooling 200 °C	0.16	0.17	0.20
	Cooling 100 °C	0.10	0.11	0.14
Na-$Ti_3C_2T_x$@Kevlar	Heating 100 °C	0.38	0.36	0.37
	Heating 200 °C	0.47	0.46	0.41
	Heating 300 °C	0.48	0.44	0.44
	Heating 400 °C	0.50	0.48	0.45
	Cooling 300 °C	0.49	0.50	0.44
	Cooling 200 °C	0.46	0.47	0.44
	Cooling 100 °C	0.43	0.44	0.38
PEEK	Heating 100 °C	0.87	-	-
	Heating 200 °C	0.95	-	-
	Heating 300 °C	1.01	-	-
	Cooling 200 °C	0.87	-	-
	Cooling 100 °C	0.90	-	-
Kevlar fabric	Heating 100 °C	0.94	-	-
	Heating 200 °C	1.10	-	-
	Heating 300 °C	1.02	-	-
	Heating 400 °C	0.93	-	-
	Cooling 300 °C	0.99	-	-
	Cooling 200 °C	0.96	-	-
	Cooling 100 °C	0.92	-	-

Supplementary Table 7. Performance summary of Na-$Ti_3C_3T_x$@Kevlar and comparison with reported work.

Coating/polymer substrate	Highest $T_{radiation}$ (°C)	$T_{radiation}$-$T_{reduction}$ (°C)	Highest $T_{Joule\ heating}$ (°C)	Washing durability R_0/R_{1h}	Bending durability R_0/R_{2000c}	Ref
$Ti_3C_2T_x$-PDA/Cotton	-	-	100.8, 8 V	-	-	38
$Ti_3C_2T_x$/ANF	100	42	63.1, 10 V	-	-	39
$Ti_3C_2T_x$/PVA/PCC	100.7	40.8	122.9, 3V	-	-	40
$Ti_3C_2T_x$/PET	150	102	/	-	-	41
$Ti_3C_2T_x$/Silk	/	/	74.6, 13 V	-	-	42
$Ti_3C_2T_x$-ITO/PET	100	59.9	106.7, 6 V	-	-	43
$Ti_3C_2T_x$/CNF	114	72.1	-	-	-	44
$Ti_3C_2T_x$/Cellulose	-	-	-	0.99 (Ω cm^{-1})/(Ω cm^{-1})	-	35
$Ti_3C_2T_x$/Cellulose	-	-	-	0.98 (Ω)/(Ω)	-	45
Nanocellulose-$Ti_3C_2T_x$/Cellulose/silicone	-	-	-	-	0.62 (Ω)/(Ω)	46
**Na-$Ti_3C_2T_x$@Kevlar**	**400**	**250**	**192.9**	**0.89**	**0.325**	**This work**

Supplementary Table 8. Lennard-Jones potential parameters and charges of water, NaCl, PDMS, and MXene.

	Water		NaCl	
	O_{water}	H_{water}	Na	Cl
σ (kcal mol^{-1})	0.1553	0.0000	0.1076	0.1004
ε (Å)	3.1660	0.0000	2.3100	4.3000
q (e)	-0.8476	+0.4238	+1.0000	-1.0000
	PDMS			
	Si	O	C	H
σ (kcal mol^{-1})	0.2443	0.2004	0.0847	0.0098
ε (Å)	3.8140	2.8200	3.4180	2.9720
q (e)	+0.7608	-0.4620	-0.5604	+0.1370
	MXene			
	Ti_{inner}	Ti_{outer}	C	O
σ (kcal mol^{-1})	0.6087	0.6087	0.0660	0.1554
ε (Å)	1.9565	1.9565	3.5000	3.1656
q (e)	0.6800	1.0400	-0.7400	-0.6400

1 Shekhirev, M., Shuck, C. E., Sarycheva, A. & Gogotsi, Y. Characterization of MXenes at every step, from their precursors to single flakes and assembled films. *Prog.Mater. Sci.* **120**, 100757 (2021).
2 Lipatov, A. *et al.* Elastic properties of 2D $Ti_3C_2T_x$ MXene monolayers and bilayers. *Sci. Adv.* **4**, eaat0491 (2018).
3 https://www.makeitfrom.com/.
4 https://www.accudynetest.com/polytable_03.html?sortby=contact_angle.
5 Jun, B.-M., Jang, M., Park, C. M., Han, J. & Yoon, Y. Selective adsorption of Cs^+ by MXene ($Ti_3C_2T_x$) from model low-level radioactive wastewater. *Nucl. Eng. Technol.* **52**, 1201-1207 (2020).
6 Ghidiu, M. *et al.* Ion-exchange and cation solvation reactions in Ti_3C_2 MXene. *Chem. Mater.* **28**, 3507-3514 (2016).
7 Pazniak, H. *et al.* Ion implantation as an approach for structural modifications and functionalization of $Ti_3C_2T_x$ MXenes. *ACS Nano* **15**, 4245-4255 (2021).
8 Sarycheva, A. & Gogotsi, Y. Raman spectroscopy analysis of the structure and surface chemistry of $Ti_3C_2T_x$ MXene. *Chem. Mater.* **32**, 3480-3488 (2020).
9 Wang, X., Wang, Z. & Qiu, J. Stabilizing MXene by hydration chemistry in aqueous solution. *Angew. Chem.* **133**, 26791-26795 (2021).
10 Mathis, T. S. *et al.* Modified MAX phase synthesis for environmentally stable and highly conductive Ti_3C_2 MXene. *ACS Nano* **15**, 6420-6429 (2021).
11 Zhang, J. *et al.* Scalable manufacturing of free-standing, strong $Ti_3C_2T_x$ MXene films with outstanding conductivity. *Adv. Mater.* **32**, 2001093 (2020).
12 Ling, Z. *et al.* Flexible and conductive MXene films and nanocomposites with high capacitance. *Proc. Nat. Acad. Sci. U.S.A.* **111**, 16676-16681 (2014).
13 Chen, H. *et al.* Pristine titanium carbide MXene films with environmentally stable conductivity and superior mechanical strength. *Adv. Funct. Mater.* **30**, 1906996 (2020).
14 Lipton, J. *et al.* Scalable, highly conductive, and micropatternable MXene films for enhanced electromagnetic interference shielding. *Matter* **3**, 546-557 (2020).
15 Kim, S. J. *et al.* Interfacial assembly of ultrathin, functional MXene films. *ACS Appl. Mater. Interfaces* **11**, 32320-32327 (2019).
16 Wan, S. *et al.* High-strength scalable MXene films through bridging-induced densification. *Science* **374**, 96-99 (2021).
17 Liu, J. *et al.* Ultrastrong and highly conductive MXene-based films for high-performance electromagnetic interference shielding. *Adv. Electron. Mater.* **6**, 1901094 (2020).
18 Cao, W.-T. *et al.* Binary strengthening and toughening of MXene/cellulose nanofiber composite paper with nacre-inspired structure and superior electromagnetic interference shielding properties. *ACS Nano* **12**, 4583-4593 (2018).
19 Lee, G. S. *et al.* Mussel inspired highly aligned $Ti_3C_2T_x$ MXene film with synergistic enhancement of mechanical strength and ambient stability. *ACS Nano* **14**, 11722-11732 (2020).
20 Tian, W. *et al.* Multifunctional nanocomposites with high strength and capacitance using 2D MXene and 1D nanocellulose. *Adv. Mater.* **31**, 1902977 (2019).
21 Wang, J., Ma, X., Zhou, J., Du, F. & Teng, C. Bioinspired, high-strength, and flexible MXene/aramid fiber for electromagnetic interference shielding papers with joule heating performance. *ACS Nano* **16**, 6700-6711 (2022).
22 Liu, Z. *et al.* Electrically conductive aluminum ion-reinforced MXene films for efficient electromagnetic interference shielding. *J. Mater. Chem. C* **8**, 1673-1678 (2020).
23 Sarycheva, A. *et al.* 2D titanium carbide (MXene) for wireless communication. *Sci. Adv.* **4**, eaau0920 (2018).

24 Liao, L., Jiang, D., Zheng, K., Zhang, M. & Liu, J. Industry-scale and environmentally stable $Ti_3C_2T_x$ MXene based film for flexible energy storage devices. *Adv. Funct. Mater.* **31**, 2103960 (2021).
25 Zhang, C. *et al.* Transparent, flexible, and conductive 2D titanium carbide (MXene) films with high volumetric capacitance. *Adv. Mater.* **29**, 1702678 (2017).
26 Han, M. *et al.* Solution-processed $Ti_3C_2T_x$ MXene antennas for radio-frequency communication. *Adv. Mater.* **33**, 2003225 (2021).
27 Yu, J. *et al.* Protein-induced decoration of applying MXene directly to UHMWPE fibers and fabrics for improved adhesion properties and electronic textiles. *Compos. Sci. Technol.* **218**, 109158 (2022).
28 Rasool, K. *et al.* Efficient antibacterial membrane based on two-dimensional $Ti_3C_2T_x$ (MXene) nanosheets. *Sci.Rep.* **7**, 1598 (2017).
29 Park, T. H. *et al.* Shape-adaptable 2D titanium carbide (MXene) heater. *ACS Nano* **13**, 6835-6844 (2019).
30 Zhou, B. *et al.* Flexible MXene/silver nanowire-based transparent conductive film with electromagnetic interference shielding and electro-photo-thermal performance. *ACS Appl. Mater. Interfaces* **12**, 40859-40869 (2020).
31 Wang, X. *et al.* A lightweight MXene-coated nonwoven fabric with excellent flame retardancy, EMI shielding, and electrothermal/photothermal conversion for wearable heater. *Chem. Eng. J.* **430**, 132605 (2022).
32 Xu, M.-K. *et al.* Electrically conductive $Ti_3C_2T_x$ MXene/polypropylene nanocomposites with an ultralow percolation threshold for efficient electromagnetic interference shielding. *Ind. Eng. Chem. Res.* **60**, 4342-4350 (2021).
33 An, H. *et al.* Surface-agnostic highly stretchable and bendable conductive MXene multilayers. *Sci. Adv.* **4**, eaaq0118 (2018).
34 Yin, J. *et al.* MXene-based hydrogels endow polyetheretherketone with effective osteogenicity and combined treatment of osteosarcoma and bacterial infection. *ACS Appl. Mater. Interfaces* **12**, 45891-45903 (2020).
35 Uzun, S. *et al.* Knittable and washable multifunctional MXene-coated cellulose yarns. *Adv. Funct. Mater.* **29**, 1905015 (2019).
36 Levitt, A. *et al.* 3D knitted energy storage textiles using MXene-coated yarns. *Mater. Today* **34**, 17-29 (2020).
37 Zeng, Z.-H. *et al.* Porous and ultra-flexible crosslinked MXene/polyimide composites for multifunctional electromagnetic interference shielding. *Nano-micro Lett.* **14**, 59 (2022).
38 Yan, B. *et al.* Orderly self-stacking a high-stability coating of MXene@polydopamine hybrid onto textiles for multifunctional personal thermal management. *Compos. Part A Appl. Sci. Manuf.* **160**, 107038 (2022).
39 Dang, W., Guo, W., Chen, W. & Zhang, Q. Tailoring of a robust asymmetric aramid nanofibers/MXene aerogel film for enhanced infrared thermal camouflage and Joule heating performances. *Nano Res.*, 1-9 (2023).
40 Li, X. *et al.* Wearable Janus-type film with integrated all-season active/passive thermal management, thermal camouflage, and ultra-high electromagnetic shielding efficiency tunable by origami process. *Adv. Funct. Mater.* **33**, 2212776 (2023).
41 Ma, H. *et al.* Blade-coated $Ti_3C_2T_x$ MXene films for pseudocapacitive energy storage and infrared stealth. *Diam. Relat. Mater.* **131**, 109587 (2023).
42 Yang, H. *et al.* Multifunctional and durable thermal management coating from sericin-MXene biohybrid on silk fabric micro-etched by deep eutectic solvent. *Appl. Surf. Sci.* **623**, 156962 (2023).

43 Du, X. *et al.* Visible transparent, infrared stealthy polymeric films with nanocoating of ITO@MXene enable efficient passive radiative heating and solar/electric thermal conversion. *Nano Res.* **16**, 3326-3332 (2023).
44 Feng, S. *et al.* Rheology-guided assembly of a highly aligned MXene/cellulose nanofiber composite film for high-performance electromagnetic interference shielding and infrared stealth. *ACS Appl. Mater. Interfaces* **14**, 36060-36070 (2022).
45 Yu, Q. *et al.* $Ti_3C_2T_x$@nonwoven fabric composite: promising MXene-coated fabric for wearable piezoresistive pressure sensors. *ACS Appl. Mater. Interfaces* **14**, 9632-9643 (2022).
46 Zhou, Z., Song, Q., Huang, B., Feng, S. & Lu, C. Facile fabrication of densely packed Ti_3C_2 MXene/nanocellulose composite films for enhancing electromagnetic interference shielding and electro-/photothermal performance. *ACS Nano* **15**, 12405-12417 (2021).
